# Supplementary material for: Multi‐dataset identification of innovative feature genes and molecular mechanisms in keratoconus
Source: J Cell Mol Med. 2024 Sep 19;28(18):e70079. doi: 10.1111/jcmm.70079 (PMC11412914; doi:10.1111/jcmm.70079)
Supplement: Supplementary file 2 — Material S2. [file JCMM-28-e70079-s002.zip › 7.GSEA analysis/1.ARL11/KEGG_MAPK_SIGNALING_PATHWAY.html]

Details for gene set KEGG\_MAPK\_SIGNALING\_PATHWAY[GSEA]

|  || Dataset | ARL11.group.cls#h\_versus\_l |
| Phenotype | group.cls#h\_versus\_l |
| Upregulated in class | l |
| GeneSet | KEGG\_MAPK\_SIGNALING\_PATHWAY |
| Enrichment Score (ES) | -0.25476062 |
| Normalized Enrichment Score (NES) | -1.0615627 |
| Nominal p-value | 0.34502923 |
| FDR q-value | 1.0 |
| FWER p-Value | 0.998 |
Table: GSEA Results Summary

  

Fig 1: Enrichment plot: KEGG\_MAPK\_SIGNALING\_PATHWAY      
 Profile of the Running ES Score & Positions of GeneSet Members on the Rank Ordered List

  

| SYMBOL | TITLE | RANK IN GENE LIST | RANK METRIC SCORE | RUNNING ES | CORE ENRICHMENT || 1 | FGF16 | na | 91 | 0.669 | 0.0227 | No |
| 2 | FGF14 | na | 927 | 0.398 | 0.0135 | No |
| 3 | CACNA1I | na | 966 | 0.393 | 0.0273 | No |
| 4 | CACNB1 | na | 1069 | 0.381 | 0.0388 | No |
| 5 | CACNG8 | na | 1424 | 0.344 | 0.0415 | No |
| 6 | CACNA1F | na | 1554 | 0.333 | 0.0504 | No |
| 7 | TGFBR2 | na | 2250 | 0.279 | 0.0408 | No |
| 8 | CACNA1A | na | 2621 | 0.259 | 0.0398 | No |
| 9 | FGF23 | na | 3267 | 0.229 | 0.0298 | No |
| 10 | CHP2 | na | 4525 | 0.182 | 0.0002 | No |
| 11 | RAC3 | na | 4612 | 0.179 | 0.0045 | No |
| 12 | NTRK1 | na | 4617 | 0.179 | 0.0111 | No |
| 13 | PLA2G4E | na | 4751 | 0.174 | 0.0138 | No |
| 14 | RASGRF2 | na | 4901 | 0.169 | 0.0159 | No |
| 15 | CACNA1B | na | 5425 | 0.154 | 0.0065 | No |
| 16 | HSPA2 | na | 5536 | 0.150 | 0.0090 | No |
| 17 | ELK1 | na | 5841 | 0.141 | 0.0055 | No |
| 18 | CACNA1H | na | 5861 | 0.141 | 0.0103 | No |
| 19 | PDGFRA | na | 5971 | 0.138 | 0.0124 | No |
| 20 | SOS1 | na | 6194 | 0.132 | 0.0109 | No |
| 21 | CACNA2D4 | na | 6234 | 0.131 | 0.0147 | No |
| 22 | PLA2G3 | na | 6310 | 0.129 | 0.0174 | No |
| 23 | FLNB | na | 6921 | 0.113 | 0.0040 | No |
| 24 | RPS6KA5 | na | 7067 | 0.110 | 0.0040 | No |
| 25 | MAP3K6 | na | 7078 | 0.110 | 0.0079 | No |
| 26 | PDGFA | na | 7165 | 0.108 | 0.0095 | No |
| 27 | PTPRR | na | 7186 | 0.108 | 0.0130 | No |
| 28 | HSPA8 | na | 7386 | 0.104 | 0.0111 | No |
| 29 | CD14 | na | 7503 | 0.102 | 0.0116 | No |
| 30 | FGF9 | na | 7521 | 0.101 | 0.0150 | No |
| 31 | TGFB2 | na | 7601 | 0.100 | 0.0164 | No |
| 32 | TAOK2 | na | 7754 | 0.097 | 0.0157 | No |
| 33 | RASA1 | na | 7808 | 0.096 | 0.0178 | No |
| 34 | FGF7 | na | 7913 | 0.094 | 0.0184 | No |
| 35 | MECOM | na | 7964 | 0.093 | 0.0204 | No |
| 36 | CACNG5 | na | 8337 | 0.086 | 0.0129 | No |
| 37 | PTPN5 | na | 8340 | 0.086 | 0.0161 | No |
| 38 | DUSP9 | na | 8469 | 0.083 | 0.0155 | No |
| 39 | NF1 | na | 8612 | 0.081 | 0.0145 | No |
| 40 | IKBKB | na | 8698 | 0.079 | 0.0150 | No |
| 41 | PPM1B | na | 8748 | 0.079 | 0.0166 | No |
| 42 | FOS | na | 8788 | 0.078 | 0.0184 | No |
| 43 | RPS6KA2 | na | 8887 | 0.077 | 0.0184 | No |
| 44 | FGF8 | na | 8904 | 0.076 | 0.0209 | No |
| 45 | CACNA2D3 | na | 8962 | 0.075 | 0.0221 | No |
| 46 | HSPA1A | na | 9156 | 0.072 | 0.0192 | No |
| 47 | PLA2G12B | na | 9215 | 0.072 | 0.0202 | No |
| 48 | FGF1 | na | 9240 | 0.071 | 0.0222 | No |
| 49 | AKT2 | na | 9348 | 0.070 | 0.0218 | No |
| 50 | ARRB1 | na | 9396 | 0.069 | 0.0230 | No |
| 51 | HSPA6 | na | 9438 | 0.068 | 0.0244 | No |
| 52 | TP53 | na | 9447 | 0.068 | 0.0267 | No |
| 53 | FGF11 | na | 9544 | 0.067 | 0.0265 | No |
| 54 | CASP3 | na | 9607 | 0.066 | 0.0272 | No |
| 55 | PRKX | na | 9610 | 0.066 | 0.0296 | No |
| 56 | FGFR3 | na | 9769 | 0.063 | 0.0274 | No |
| 57 | RASGRP2 | na | 9889 | 0.062 | 0.0263 | No |
| 58 | FGF12 | na | 9953 | 0.061 | 0.0267 | No |
| 59 | SOS2 | na | 9955 | 0.061 | 0.0290 | No |
| 60 | MAP3K3 | na | 10136 | 0.059 | 0.0260 | No |
| 61 | MYC | na | 10444 | 0.054 | 0.0192 | No |
| 62 | MAPT | na | 10591 | 0.053 | 0.0169 | No |
| 63 | PRKCA | na | 10617 | 0.052 | 0.0182 | No |
| 64 | BRAF | na | 10628 | 0.052 | 0.0199 | No |
| 65 | KRAS | na | 10630 | 0.052 | 0.0218 | No |
| 66 | HSPA1B | na | 10821 | 0.050 | 0.0182 | No |
| 67 | STK3 | na | 11080 | 0.047 | 0.0124 | No |
| 68 | EGFR | na | 11115 | 0.046 | 0.0132 | No |
| 69 | MAPK9 | na | 11167 | 0.046 | 0.0135 | No |
| 70 | MAPK11 | na | 11276 | 0.044 | 0.0120 | No |
| 71 | FGFR2 | na | 11489 | 0.042 | 0.0074 | No |
| 72 | TRAF2 | na | 11495 | 0.042 | 0.0089 | No |
| 73 | MAPK7 | na | 11510 | 0.041 | 0.0100 | No |
| 74 | MKNK2 | na | 11748 | 0.039 | 0.0046 | No |
| 75 | ELK4 | na | 11979 | 0.036 | -0.0007 | No |
| 76 | MAP3K4 | na | 12080 | 0.035 | -0.0023 | No |
| 77 | MAP3K1 | na | 12394 | 0.032 | -0.0101 | No |
| 78 | MAX | na | 12403 | 0.032 | -0.0091 | No |
| 79 | RPS6KA4 | na | 12471 | 0.032 | -0.0099 | No |
| 80 | TAOK1 | na | 12628 | 0.030 | -0.0133 | No |
| 81 | MAPK12 | na | 13078 | 0.026 | -0.0253 | No |
| 82 | NFATC2 | na | 13211 | 0.025 | -0.0282 | No |
| 83 | MKNK1 | na | 13296 | 0.025 | -0.0297 | No |
| 84 | JUN | na | 13319 | 0.024 | -0.0294 | No |
| 85 | PLA2G2C | na | 13424 | 0.024 | -0.0315 | No |
| 86 | EGF | na | 13590 | 0.022 | -0.0355 | No |
| 87 | DUSP6 | na | 13641 | 0.022 | -0.0361 | No |
| 88 | DUSP2 | na | 13850 | 0.020 | -0.0414 | No |
| 89 | STMN1 | na | 13968 | 0.019 | -0.0440 | No |
| 90 | MAP2K5 | na | 14281 | 0.017 | -0.0525 | No |
| 91 | JMJD7-PLA2G4B | na | 14509 | 0.015 | -0.0585 | No |
| 92 | MOS | na | 14635 | 0.014 | -0.0616 | No |
| 93 | MAP2K7 | na | 14659 | 0.014 | -0.0617 | No |
| 94 | FGF13 | na | 14704 | 0.013 | -0.0625 | No |
| 95 | ATF4 | na | 14783 | 0.013 | -0.0643 | No |
| 96 | HSPB1 | na | 14853 | 0.012 | -0.0658 | No |
| 97 | MAP3K11 | na | 15001 | 0.011 | -0.0697 | No |
| 98 | FGF10 | na | 15104 | 0.010 | -0.0722 | No |
| 99 | CHUK | na | 15127 | 0.010 | -0.0725 | No |
| 100 | MAPK10 | na | 15299 | 0.009 | -0.0771 | No |
| 101 | RRAS | na | 15323 | 0.009 | -0.0774 | No |
| 102 | MAPK8IP2 | na | 15366 | 0.008 | -0.0783 | No |
| 103 | FGF19 | na | 15552 | 0.007 | -0.0834 | No |
| 104 | NLK | na | 15607 | 0.007 | -0.0847 | No |
| 105 | MAPKAPK3 | na | 15664 | 0.006 | -0.0861 | No |
| 106 | DUSP3 | na | 15706 | 0.006 | -0.0871 | No |
| 107 | FASLG | na | 15958 | 0.004 | -0.0942 | No |
| 108 | RAC1 | na | 16390 | 0.001 | -0.1067 | No |
| 109 | NTF4 | na | 16572 | -0.000 | -0.1119 | No |
| 110 | RPS6KA3 | na | 16678 | -0.001 | -0.1149 | No |
| 111 | HSPA1L | na | 16824 | -0.002 | -0.1191 | No |
| 112 | MAP2K2 | na | 16997 | -0.003 | -0.1239 | No |
| 113 | PLA2G4A | na | 17245 | -0.005 | -0.1309 | No |
| 114 | NR4A1 | na | 17280 | -0.005 | -0.1317 | No |
| 115 | PRKCG | na | 17545 | -0.007 | -0.1391 | No |
| 116 | MAPK14 | na | 17567 | -0.007 | -0.1394 | No |
| 117 | PLA2G6 | na | 17692 | -0.008 | -0.1427 | No |
| 118 | MAP2K4 | na | 17706 | -0.008 | -0.1428 | No |
| 119 | RAP1B | na | 17707 | -0.008 | -0.1425 | No |
| 120 | RAPGEF2 | na | 17786 | -0.009 | -0.1444 | No |
| 121 | PLA2G12A | na | 17822 | -0.009 | -0.1451 | No |
| 122 | AKT1 | na | 18085 | -0.011 | -0.1523 | No |
| 123 | TNFRSF1A | na | 18347 | -0.013 | -0.1594 | No |
| 124 | CDC42 | na | 18361 | -0.013 | -0.1593 | No |
| 125 | HRAS | na | 18435 | -0.013 | -0.1609 | No |
| 126 | CACNG3 | na | 18568 | -0.014 | -0.1642 | No |
| 127 | ARRB2 | na | 18772 | -0.015 | -0.1695 | No |
| 128 | FGF3 | na | 18816 | -0.016 | -0.1701 | No |
| 129 | RAC2 | na | 18868 | -0.016 | -0.1710 | No |
| 130 | FGFR4 | na | 18927 | -0.016 | -0.1721 | No |
| 131 | PRKACB | na | 18969 | -0.017 | -0.1726 | No |
| 132 | MEF2C | na | 18982 | -0.017 | -0.1723 | No |
| 133 | STK4 | na | 18997 | -0.017 | -0.1721 | No |
| 134 | RAP1A | na | 18999 | -0.017 | -0.1715 | No |
| 135 | PPP3R1 | na | 19029 | -0.017 | -0.1717 | No |
| 136 | TAB1 | na | 19054 | -0.017 | -0.1717 | No |
| 137 | DAXX | na | 19077 | -0.017 | -0.1717 | No |
| 138 | TAOK3 | na | 19187 | -0.018 | -0.1742 | No |
| 139 | CACNB4 | na | 19492 | -0.020 | -0.1822 | No |
| 140 | PPP3CA | na | 19531 | -0.021 | -0.1825 | No |
| 141 | CACNA1D | na | 19640 | -0.021 | -0.1849 | No |
| 142 | TGFBR1 | na | 19919 | -0.023 | -0.1921 | No |
| 143 | GADD45B | na | 19948 | -0.023 | -0.1920 | No |
| 144 | PLA2G2E | na | 19951 | -0.023 | -0.1912 | No |
| 145 | MAP2K3 | na | 20612 | -0.028 | -0.2092 | No |
| 146 | MAPK8IP1 | na | 20893 | -0.030 | -0.2162 | No |
| 147 | CDC25B | na | 20940 | -0.031 | -0.2164 | No |
| 148 | MAPK13 | na | 20970 | -0.031 | -0.2160 | No |
| 149 | TRAF6 | na | 21028 | -0.032 | -0.2165 | No |
| 150 | CHP1 | na | 21036 | -0.032 | -0.2155 | No |
| 151 | PPP3CB | na | 21103 | -0.032 | -0.2162 | No |
| 152 | MAPK8IP3 | na | 21106 | -0.032 | -0.2151 | No |
| 153 | RASGRP1 | na | 21451 | -0.035 | -0.2237 | No |
| 154 | MAP3K13 | na | 21527 | -0.035 | -0.2246 | No |
| 155 | MAPKAPK5 | na | 21840 | -0.038 | -0.2322 | No |
| 156 | FGF22 | na | 22003 | -0.040 | -0.2354 | No |
| 157 | RPS6KA1 | na | 22083 | -0.041 | -0.2361 | No |
| 158 | MAP3K7 | na | 22108 | -0.041 | -0.2353 | No |
| 159 | CACNB2 | na | 22149 | -0.041 | -0.2349 | No |
| 160 | ECSIT | na | 22178 | -0.042 | -0.2341 | No |
| 161 | CACNG1 | na | 22207 | -0.042 | -0.2333 | No |
| 162 | JUND | na | 22242 | -0.042 | -0.2327 | No |
| 163 | FGF21 | na | 22495 | -0.045 | -0.2383 | No |
| 164 | RELB | na | 22538 | -0.045 | -0.2378 | No |
| 165 | DUSP1 | na | 22561 | -0.046 | -0.2367 | No |
| 166 | MAPK3 | na | 22614 | -0.046 | -0.2365 | No |
| 167 | TNF | na | 22702 | -0.047 | -0.2372 | No |
| 168 | GNG12 | na | 22709 | -0.047 | -0.2356 | No |
| 169 | IKBKG | na | 22799 | -0.048 | -0.2364 | No |
| 170 | GRB2 | na | 23043 | -0.051 | -0.2415 | No |
| 171 | MAP4K1 | na | 23227 | -0.053 | -0.2448 | No |
| 172 | FGF18 | na | 23239 | -0.053 | -0.2432 | No |
| 173 | RASGRP4 | na | 23303 | -0.053 | -0.2430 | No |
| 174 | RAF1 | na | 23336 | -0.054 | -0.2419 | No |
| 175 | CACNG2 | na | 23379 | -0.054 | -0.2410 | No |
| 176 | NFATC4 | na | 23442 | -0.055 | -0.2407 | No |
| 177 | DUSP8 | na | 23584 | -0.057 | -0.2427 | No |
| 178 | PAK1 | na | 23649 | -0.058 | -0.2423 | No |
| 179 | PRKACA | na | 23665 | -0.058 | -0.2406 | No |
| 180 | PPM1A | na | 23667 | -0.058 | -0.2384 | No |
| 181 | RASGRF1 | na | 23766 | -0.059 | -0.2390 | No |
| 182 | PLA2G10 | na | 23788 | -0.060 | -0.2374 | No |
| 183 | TAB2 | na | 23865 | -0.060 | -0.2373 | No |
| 184 | RPS6KA6 | na | 24013 | -0.062 | -0.2392 | No |
| 185 | FAS | na | 24099 | -0.064 | -0.2392 | No |
| 186 | FLNA | na | 24142 | -0.064 | -0.2380 | No |
| 187 | LAMTOR3 | na | 24503 | -0.069 | -0.2458 | No |
| 188 | MAPK8 | na | 24525 | -0.070 | -0.2438 | No |
| 189 | MAPK1 | na | 24626 | -0.071 | -0.2440 | No |
| 190 | MAP3K12 | na | 24789 | -0.073 | -0.2459 | No |
| 191 | PAK2 | na | 24900 | -0.075 | -0.2463 | No |
| 192 | RRAS2 | na | 25031 | -0.076 | -0.2472 | No |
| 193 | CACNA1C | na | 25293 | -0.080 | -0.2517 | Yes |
| 194 | NTRK2 | na | 25301 | -0.080 | -0.2489 | Yes |
| 195 | GNA12 | na | 25331 | -0.080 | -0.2467 | Yes |
| 196 | GADD45G | na | 25342 | -0.081 | -0.2440 | Yes |
| 197 | TGFB1 | na | 25459 | -0.082 | -0.2442 | Yes |
| 198 | CRK | na | 25703 | -0.086 | -0.2480 | Yes |
| 199 | PRKACG | na | 25752 | -0.087 | -0.2461 | Yes |
| 200 | MAPKAPK2 | na | 25894 | -0.089 | -0.2469 | Yes |
| 201 | FGF20 | na | 25897 | -0.089 | -0.2436 | Yes |
| 202 | MAP3K2 | na | 26106 | -0.092 | -0.2461 | Yes |
| 203 | PPP5C | na | 26120 | -0.092 | -0.2430 | Yes |
| 204 | CACNA2D2 | na | 26358 | -0.096 | -0.2463 | Yes |
| 205 | PPP3R2 | na | 26430 | -0.097 | -0.2447 | Yes |
| 206 | GADD45A | na | 26446 | -0.098 | -0.2414 | Yes |
| 207 | DUSP14 | na | 26454 | -0.098 | -0.2379 | Yes |
| 208 | NTF3 | na | 26592 | -0.100 | -0.2381 | Yes |
| 209 | MAP3K5 | na | 26641 | -0.101 | -0.2356 | Yes |
| 210 | DUSP16 | na | 26669 | -0.102 | -0.2326 | Yes |
| 211 | CACNB3 | na | 26714 | -0.103 | -0.2300 | Yes |
| 212 | NFKB1 | na | 26767 | -0.104 | -0.2275 | Yes |
| 213 | ATF2 | na | 27096 | -0.110 | -0.2329 | Yes |
| 214 | AKT3 | na | 27547 | -0.120 | -0.2414 | Yes |
| 215 | NRAS | na | 27790 | -0.126 | -0.2437 | Yes |
| 216 | MAP4K2 | na | 27810 | -0.126 | -0.2395 | Yes |
| 217 | MAP4K4 | na | 27811 | -0.126 | -0.2347 | Yes |
| 218 | CACNG7 | na | 27851 | -0.127 | -0.2310 | Yes |
| 219 | CRKL | na | 27878 | -0.128 | -0.2269 | Yes |
| 220 | PPP3CC | na | 27901 | -0.128 | -0.2227 | Yes |
| 221 | MAP3K8 | na | 27945 | -0.129 | -0.2191 | Yes |
| 222 | PLA2G2D | na | 27985 | -0.130 | -0.2153 | Yes |
| 223 | NFKB2 | na | 28308 | -0.139 | -0.2194 | Yes |
| 224 | MAP3K14 | na | 28361 | -0.140 | -0.2156 | Yes |
| 225 | IL1B | na | 28530 | -0.144 | -0.2150 | Yes |
| 226 | IL1A | na | 28564 | -0.145 | -0.2105 | Yes |
| 227 | RASA2 | na | 28740 | -0.150 | -0.2098 | Yes |
| 228 | MAP2K6 | na | 28948 | -0.155 | -0.2100 | Yes |
| 229 | MAP4K3 | na | 29007 | -0.156 | -0.2057 | Yes |
| 230 | RELA | na | 29186 | -0.162 | -0.2048 | Yes |
| 231 | DUSP4 | na | 29297 | -0.166 | -0.2017 | Yes |
| 232 | SRF | na | 29318 | -0.166 | -0.1960 | Yes |
| 233 | FGF6 | na | 29398 | -0.169 | -0.1919 | Yes |
| 234 | PDGFRB | na | 29523 | -0.173 | -0.1889 | Yes |
| 235 | CACNG4 | na | 29566 | -0.174 | -0.1836 | Yes |
| 236 | CACNA2D1 | na | 29712 | -0.179 | -0.1810 | Yes |
| 237 | FLNC | na | 30076 | -0.190 | -0.1843 | Yes |
| 238 | BDNF | na | 30179 | -0.194 | -0.1800 | Yes |
| 239 | IL1R1 | na | 30266 | -0.196 | -0.1750 | Yes |
| 240 | DUSP7 | na | 30546 | -0.206 | -0.1754 | Yes |
| 241 | IL1R2 | na | 30605 | -0.208 | -0.1692 | Yes |
| 242 | PLA2G2F | na | 30623 | -0.208 | -0.1618 | Yes |
| 243 | CACNA1E | na | 31035 | -0.222 | -0.1653 | Yes |
| 244 | MAP2K1 | na | 31070 | -0.223 | -0.1579 | Yes |
| 245 | DDIT3 | na | 31332 | -0.233 | -0.1566 | Yes |
| 246 | NGF | na | 31452 | -0.237 | -0.1511 | Yes |
| 247 | PTPN7 | na | 31489 | -0.239 | -0.1431 | Yes |
| 248 | FGF5 | na | 31584 | -0.243 | -0.1366 | Yes |
| 249 | MRAS | na | 31743 | -0.250 | -0.1318 | Yes |
| 250 | CACNG6 | na | 31816 | -0.253 | -0.1243 | Yes |
| 251 | PLA2G5 | na | 32093 | -0.264 | -0.1223 | Yes |
| 252 | DUSP5 | na | 32202 | -0.269 | -0.1153 | Yes |
| 253 | PLA2G2A | na | 32310 | -0.275 | -0.1080 | Yes |
| 254 | FGF17 | na | 32313 | -0.275 | -0.0976 | Yes |
| 255 | PDGFB | na | 32445 | -0.283 | -0.0907 | Yes |
| 256 | FGF2 | na | 33068 | -0.326 | -0.0964 | Yes |
| 257 | TGFB3 | na | 33262 | -0.342 | -0.0890 | Yes |
| 258 | PLA2G1B | na | 33526 | -0.362 | -0.0830 | Yes |
| 259 | DUSP10 | na | 33583 | -0.367 | -0.0707 | Yes |
| 260 | RASGRP3 | na | 33816 | -0.391 | -0.0626 | Yes |
| 261 | PRKCB | na | 33863 | -0.399 | -0.0489 | Yes |
| 262 | CACNA1G | na | 33871 | -0.400 | -0.0339 | Yes |
| 263 | CACNA1S | na | 33887 | -0.403 | -0.0191 | Yes |
| 264 | FGFR1 | na | 34437 | -0.510 | -0.0157 | Yes |
| 265 | FGF4 | na | 34644 | -0.638 | 0.0024 | Yes |
Table: GSEA details [plain text format]

  

Fig 2: KEGG\_MAPK\_SIGNALING\_PATHWAY      
 Blue-Pink O' Gram in the Space of the Analyzed GeneSet

  

Fig 3: KEGG\_MAPK\_SIGNALING\_PATHWAY: Random ES distribution      
 Gene set null distribution of ES for **KEGG\_MAPK\_SIGNALING\_PATHWAY**
